# Thermal-Recoverable Tough Hydrogels Enhanced by Porphyrin Decorated Graphene Oxide

**DOI:** 10.3390/nano9101487

**Published:** 2019-10-18

**Authors:** Jilong Wang, Junhua Wei, Siheng Su, Jingjing Qiu, Zhonglue Hu, Molla Hasan, Evan Vargas, Michelle Pantoya, Shiren Wang

**Affiliations:** 1 Key Laboratory of Textile Science & Technology of Ministry of Education, College of Textiles, Donghua University, Shanghai 201620, China; jilong.wang@dhu.edu.cn; 2 Department of Mechanical Engineering, Texas Tech University, 2500 Broadway, P.O. Box 43061, Lubbock, TX 79409, USA; evan.vargas@ttu.edu (E.V.); michelle.pantoya@ttu.edu (M.P.); 3 Department of Mechanical Engineering, California State University at Fullerton, Fullerton, CA 92831, USA; ssu@fullerton.edu; 4 College of Engineering, Zhejiang Normal University, Jinhua 321000, China; zhonglue.hu@zjnu.edu.cn; 5 Inamori School of Engineering, Alfred University, Alfred, NY 14802, USA; hasanm@alfred.edu; 6 Department of Industrial and Systems Engineering, Texas A&M University, College Station, TX 77843, USA; s.wang@tamu.edu

**Keywords:** porphyrin decorated graphene oxide, hydrogels, double network, photothermal recovery, compressive strength

## Abstract

Artificial tissue materials usually suffer properties and structure loss over time. As a usual strategy, a new substitution is required to replace the worn one to maintain the functions. Although several approaches have been developed to restore the mechanical properties of hydrogels, they require direct heating or touching, which cannot be processed within the body. In this manuscript, a photothermal method was developed to restore the mechanical properties of the tough hydrogels by using near infrared (NIR) laser irradiation. By adding the porphyrin decorated graphene oxide (PGO) as the nanoreinforcer and photothermal agent into carrageenan/polyacrylamide double network hydrogels (PDN), the compressive strength of the PDN was greatly improved by 104%. Under a short time of NIR laser irradiation, the PGO effectively converts light energy to thermal energy to heat the PDN hydrogels. The damaged carrageenan network was rebuilt, and a 90% compressive strength recovery was achieved. The PGO not only significantly improves the mechanical performance of PDN, but also restores the compressive property of PDN via a photothermal method. These tough hydrogels with superior photothermal recovery may work as promising substitutes for load-bearing tissues.

## 1. Introduction

Hydrogels are three-dimensional networks composed of a high-molecular-weight polymer, water, and cross-linker, which have been employed as scaffolds in tissue engineering [1,2,3], carriers for drug delivery [4,5], and super-absorbents in disposable products [6,7]. However, the practical applications as artificial load-bearing soft tissues are still strikingly challenged because of their soft nature. Tremendous attempts have been made to develop mechanical-superior hydrogels, like double network (DN) hydrogels, slide-ring hydrogels, nanocomposite hydrogels, triblock copolymers hydrogels, hydrophobic modified hydrogels, tetra-PEG gels, and macromolecular microsphere composite (MMC) hydrogels [8,9,10,11,12,13,14,15,16,17,18]. Among them, double network hydrogels have been demonstrated as a superior choice, as it can achieve balanced yet improved mechanical properties between stiffness and toughness by arranging “soft” and “brittle” networks [8,19]. However, double network hydrogels, which use sacrificial bonds to achieve high strength, possess inferior fatigue resistance under continuous mechanical loading-unloading cycles [13]. This drawback severely limits the development of DN hydrogels as potential substitutes for load-bearing tissues, like cartilage. Chen et al. [8] achieved highly mechanical and thermal-recoverable DN hydrogels via introducing a thermoreversible sol-gel polysaccharide. Tong et al. [20] developed near infrared (NIR)-responsible self-healing hydrogels via introducing graphene oxide (GO)-Hectorite clay into poly(*N,N*-dimethylacrylamide) hydrogels. However, achieving hydrogels with high mechanical properties and fast self-healing simultaneously is still a challenge for practical application [20]. 

Carrageenan, a family of linear sulfated polysaccharides extracted from red seaweeds, has the ability to form thermoreversible hydrogels, which is used far more extensively than agar as an emulsifier, gelling, thickening and stabilizing agent in food and pharmaceutical industries because of their relatively low cost [21,22,23]. Most importantly, the sol-gel transition temperature of kappa-carrageenan is much lower than agar, which is slightly higher than body temperature [24,25]. These advantages make carrageenan a potential candidate in the photothermal reversible and tough gels for bio-applications. 

Diverse carbon-based materials have received considerable interest for potential applications from electronic devices to the biomedical field [26,27,28,29,30]. Due to large surface area, outstanding mechanical properties, and eminent thermal and chemical stability, graphene and its derivatives are widely used in composite materials as reinforcing fillers to improve mechanical properties and other specific functions [31,32,33,34]. By adding biocompatible graphene oxide into the carrageenan hydrogels [35,36], their mechanical properties are improved significantly as the stress is transported to the super-tough graphene oxide [37,38]. In addition, the photothermal effect of GO has been widely investigated in recent years, which efficiently convert light energy to thermal energy [39]. In our previous study [40], biocompatible porphyrin immobilized graphene oxide (PGO) has been successfully developed, which can achieve high photothermal conversion under 808 nm laser irradiation.

In this manuscript, biocompatible porphyrin decorated graphene oxide (PGO) is first introduced into the double network hydrogels to enhance the mechanical properties and possess NIR photothermal recovery. The PGO efficiently transfers the NIR light energy into thermal energy to adopt random coils in the sol state under 808 nm NIR irradiation. Upon cooling, the sacrificial network, double helix structures in carrageenan, is reformed, and approximately 90% compressive strength of the PGO reinforced carrageenan/polyacrylamide double network (PDN) hydrogels is recovered. These PDN gels exhibit improved mechanical properties and superior photothermal recovery compared to the previous double network hydrogels, which may find potential applications as promising substitutes for load-bearing tissues. 

## 2. Experimental Section

### 2.1. Materials

Graphite was provided from Asbury Carbons. Carrageen kappa, acrylamide (AAm), tetrabutylammonium hydroxide solution (TBA), 1-methyl-2-pyrrolidinone (NMP), *N*,*N*′-methylenebis (acrylamide) (MBAA), and ammonium persulfate (APS) were purchased from Sigma Aldrich, (St. Louis, MO, USA). Porphine (meso-Tetra[4-(allyloxy)phenyl] porphine chloride) was purchased from Frontier Scientific, (Logan, UT, USA).

### 2.2. Graphene Oxide (GO) Preparation

Graphite oxide was prepared according to the modified Brodie’s method [40]. Specifically, 2 g of graphite was blended with 17 g of NaClO_3_, then 80 mL of HNO_3_ was added slowly to avoid burning. After stirring at 60 °C for 6 h and then stirring for another 12 h at room temperature, the diluted HCl solution and deionized (DI) water were used to wash the mixture solution to remove impurities, separately. The exfoliated GO was collected by centrifugation at 5000 rpm for 30 min to remove the large sediment and then dialyzed by using 12,000 Da membranes in DI water for 1 week for purification.

### 2.3. Porphyrin Decorated Graphene Oxide (PGO) Preparation

**Scheme 1:**
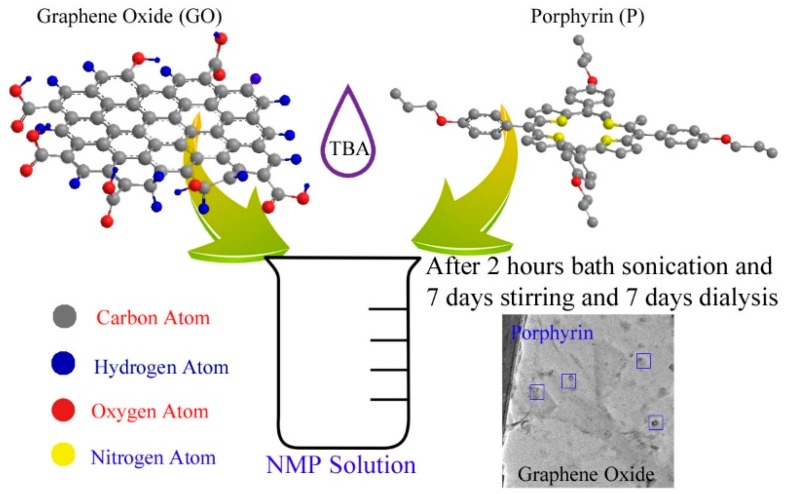
Schematic diagram of porphyrin decorated graphene oxide (PGO) fabrication.

PGO was achieved by introducing porphine onto GO through π-π interaction [41]. As shown in Scheme 1, 0.1 mg/mL graphite oxide and 0.1 mg/mL porphyrin was dispersed in 10 mL NMP and 2 mL TBA by sonication. The suspensions were stirred for 1 week to allow the attachment of porphyrin onto the GO nanosheets. Then, the black suspension was dialyzed by using 12,000 Da membranes in DI water for 1 week to remove the NMP and unattached porphyrin.

### 2.4. Hydrogels Fabrication

The PGO reinforced carrageenan/polyacrylamide (carr/PAAm) DN (PDN) hydrogels were fabricated via a two-step polymerization method [42]. After 400 mg of carrageen kappa, 2400 mg of AAm, 6 mg of MBAA (0.0025 of AAm) were dissolved into 10 mL 0.1 mg/mL PGO solution with 90 °C oil bath, and the mixture solution was injected into Teflon molds. After cooling down in the refrigerator overnight, the formed gels were immersed into 30 mL DI water with 7200 mg of AAm, 36 mg of the MBAA, and 216 mg of APS for 24 h. After removing the soft PAAm single network (SN) hydrogels, the PDN hydrogels were achieved for the following characterizations. The carr/PAAm DN hydrogels and GO reinforced carr/PAAm DN (GDN) hydrogels were prepared via following the same procedure with pure DI water and 0.1 mg/mL GO solution.

### 2.5. Mechanical Recovery of Hydrogels

The thermal recovery process is implemented as follows: after compression, hydrogels were heated at 60 °C for 5 min and cooled to room temperature. After thermal recovery, the compressive properties of hydrogels were tested again by unconfined compression. In addition, a NIR Laser generator (Dragon Lasers, Changchun, China) was used to provide NIR light. The heating rates of Control, GDN, and PDN were measured for 30 s using a thermometer. The photothermal recovery process was implemented as follows: after compression, hydrogels were heated under an 808 nm laser generator (for 10 min and cooled down to room temperature. After that, the compressive properties of hydrogels were tested again by unconfined compression. The temperature changes of DN and PDN gels were also measured by a FLIR SC8303 high-speed infrared camera. The camera was placed directly in front of DN and PDN hydrogels, recording two-dimensional transient temperature distribution images. The viewing area of the samples and holder was set at a 640 × 360 pixel window frame size, with an acquisition rate of 2 frames per second.

### 2.6. Materials Characterization

The morphology of the PGO and GO was investigated by transmission electron microscopy (TEM, HITACHI 8100, Hitachi Appliances Inc., Tokyo, Japan, and the composition of the PGO, GO, and porphyrin was tested by Fourier transform infrared spectroscopy (FTIR, Nicolet IS10 FTIR spectrometer from Thermo Scientific, Waltham, MA, USA), thermogravimetric analysis (TGA, Q50 from TA Instruments Inc., New Castle, DE, USA) and UV-vis spectrum (InfiniteM1000 Pro plate reader from Tecan, Männedorf, Switzerland). The gel-sol transition temperature of carrageen was obtained by differential scanning calorimetry (DSC, Q20 from TA Instruments Inc., New Castle, DE, USA).

### 2.7. Compressive Measurement

The mechanical properties of the hydrogels were investigated by unconfined compression using a commercial machine (AGS-X from SHIMADZU, Kyoto, Japan). An Instron 5966 (Instron Corporation, Canton, MA, USA) was used to measure cyclic compressive properties of hydrogels, and the compressive velocity was 600 µm/min. The compressive strain ɛ is defined as:

(1) $$ɛ\;=\;\frac{h}{h_{0}}$$

where *h* is the height during deformation, and *h*_0_ is the initial height of the gel sample. The normal compressive stress σ*_normal_* is defined as:

(2) $$\sigma_{normal}\;=\;\frac{F}{A_{0}}$$

where *F* is the applied load, and *A*_0_ is the original top surface area of samples.

The fatigue resistance of the DN and TN gels was characterized by a successive loading-unloading test. Three increasing compressive strains (30%, 60%, and 90%) were used in this experiment. The compressive toughness (*U*) is defined as:

(3) $$U\;=\;\int_{0}^{S}\frac{F}{\pi r^{2}}ds$$

where *F* is the applied force, and *s* is the corresponding displacement. The hysteresis energy (*U_hyst_*) is calculated as

(4) $$U_{hyst}=\frac{\int_{0}^{S_{loading}}Fds-\int_{0}^{S_{unloading}}Fds}{\pi r^{2}}.$$

### 2.8. Swelling Measurement

The swelling ratio (SR) was defined as:

SR = (Ws − Wo)/Wo × 100%(5)

where Ws and Wo represent the weight of hydrogels after swelling in DI water in different time periods and the weight of hydrogels before swelling, respectively.

## 3. Results and Discussions

### 3.1. Porphyrin-Graphene Oxide (PGO)

**Figure 1:**
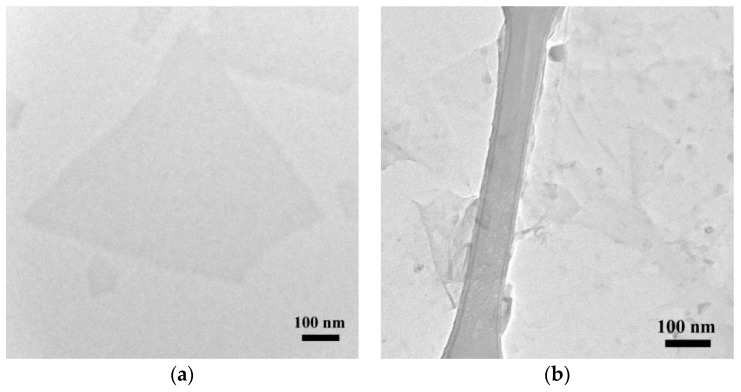
TEM images of (**a**) graphene oxide (GO) and (**b**) PGO.

The attachment of the porphyrin on the GO was confirmed by the TEM images, TGA results, FTIR spectra, zeta potential, and UV-vis spectra. TEM images showed the few-layer structures of GO (Figure 1a) and PGO (Figure 1b). Compared to clean planar GO nanosheets, a certain amount of porphyrin particle aggregations were presented on the PGO nanosheets, which clearly indicates that the GO is conjugated with porphyrin via π–π interaction. These results are similar to our previous results [40].

**Figure 2:**
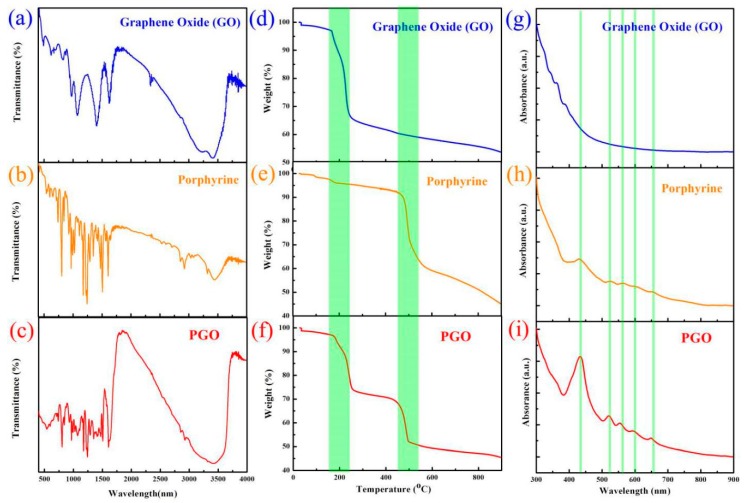
(**a**–**c**): FTIR spectra of GO, porphyrin, and PGO; (**d**–**f**): TGA curves of GO, porphyrin, and PGO; (**g**–**i**): UV-vis absorption spectra of GO, porphyrin and PGO.

Other results, including FTIR spectra, TGA curve, and UV-vis absorption, further validate the π–π interaction between porphyrin and GO in PGO, which is consistent with the TEM results. The FTIR spectra (Figure 2a–c) indicates the existence of the porphyrin on the GO by the peaks around 750–1500 nm. Figure 2a presents that the epoxy groups (1390 cm^−1^, 1060 cm^−1^, and 946 cm^−1^), carboxyl group (1720 cm^−1^), and hydroxyl group (3400 cm^−1^) existed in the as-prepared GO. However, the peaks (1720 cm^−1^, 1390 cm^−1^, 1060 cm^−1^, and 946 cm^−1^) were covered by peaks of porphyrin, and the peak of the hydroxyl group (3400 cm^−1^) did exist (Figure 2c), which demonstrates that the porphyrin was successfully combined with GO. Figure 2d–f present the TGA results of the GO, porphyrin, and PGO. Clear weight reductions around 180 °C and 400 °C were presented in GO and porphyrin, respectively, which were corresponding to the decomposition temperature of GO and porphyrin. As depicted in Figure 2f, two obvious weight cutbacks were found around 180 °C and 400 °C, which indicates that the porphyrin was attached to GO. A similar conclusion was presented in the UV-vis spectra (Figure 2g–i). No obvious peaks were shown on the spectrum of GO; however, a weak peak, called the Soret band, appeared around 420 nm on the spectrum of porphyrin, which was a result of poor solubility of porphyrin in DI water. A strong peak at the same position (420 nm) was also observed in the spectrum of PGO, which demonstrates that the porphyrin was profitably connected onto GO.

In addition, the average zeta potential of GO and PGO was around −55 mV and −25 mV, respectively (Figure S1). A slight decrease was found after GO conjugated with porphyrin, indicating that the stability of PGO resembles GO, which is also consistent with our previous results [40].

**Figure 3:**
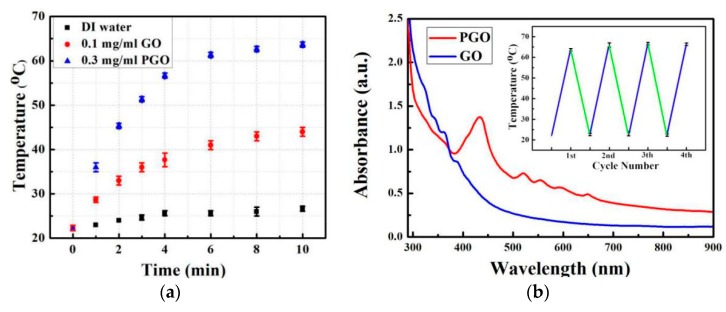
(**a**) Photothermal effects of deionized (DI) water, 0.1 mg/mL GO, and 0.1 mg/mL PGO solution with NIR laser irradiation (808 nm, 2.5 W cm^2^) for 10 min, and (**b**) UV-vis absorption spectra of GO and PGO solution at the same diluted concentration; the insert is a temperature change of 0.1 mg/mL PGO solution over four laser ON/OFF cycles (laser irradiation 10 min in every cycle).

Due to the presence of porphyrin on GO layers, it enhances the NIR adsorption and its photo-thermal conversion efficiency under NIR irradiation. As shown in Figure 3a, the temperature of DI water nearly changed. On the other hand, the 0.1 mg/mL PGO solution showed a higher temperature increase (ΔT = 41.6 °C) than the 0.1 mg/mL GO solution (ΔT = 21.7 °C), which suggests that 91.7% more heat was converted from NIR irradiation (808 nm, 2.5 W cm^2^, 10 min) by PGO compared to the GO solution. In addition, the temperature of the PGO solution was above 50 °C after 3 min of NIR laser irradiation; however, that of the GO solution was only 36 °C. These results demonstrate that PGO can efficiently adsorb NIR light and transfer it into heat energy. Figure 3b showed that the PGO solution owned higher adsorption in the NIR range, while the GO solution presented small adsorption, indicating that porphyrin conjugation enhances NIR absorbance of GO and porphyrin particle aggregations on GO nanosheets act as antennas to harvest thermal energy from NIR light. The insert in Figure 3b presented that a negligible change of temperature enhancement (ΔT ≈ 43 °C) was found after four NIR laser ON/OFF cycles, which demonstrates the stability of PGO in aqueous solution after irradiation.

### 3.2. PGO Reinforced Hydrogel (PDN)

**Figure 4:**
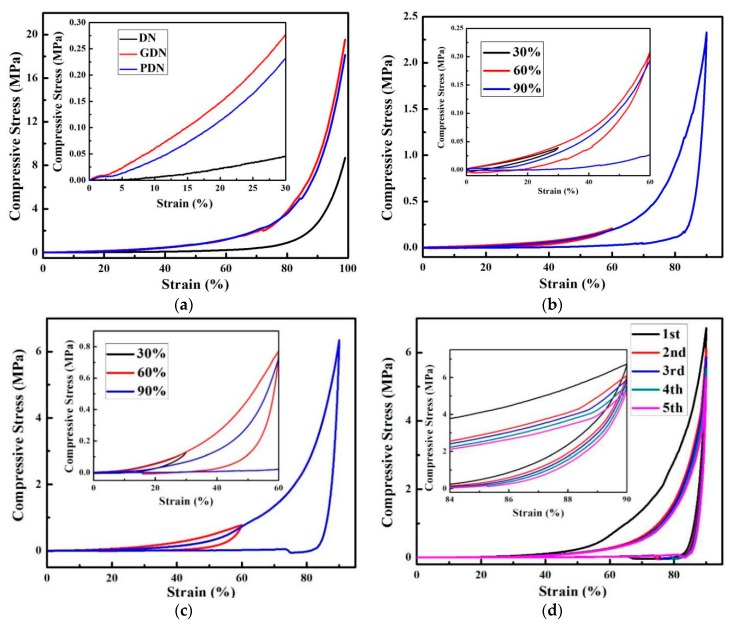

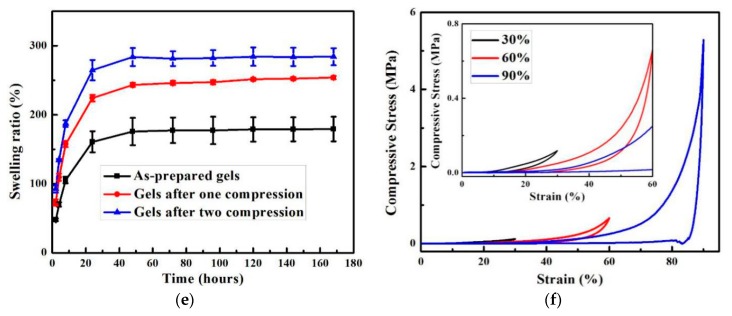
(**a**) Normal stress-strain curve of DN, GDN, and PDN hydrogels, and the typical consecutive loading-unloading curves of (**b**) DN and (**c**) PDN hydrogels with the gradient increased in maximum strain (30%, 60%, and 90%), (**d**) representative cyclic loading-unloading curves of PDN hydrogels for up to five cycles at 90% strain compression, (**e**) swelling ratio of as-prepared gels, gels after one compression, and gels after two compression, and (**f**) the hysteresis loops at the fifth loading cycle at different maximum strains of PDN gels.

The PGO reinforced DN (PDN) gels were fabricated by introducing the PGO into carr/PAAm DN hydrogels. The compressive stress-strain curves of the hydrogels were presented in Figure 4. As shown in Figure 4a and Table S1, the compressive strength was remarkably improved from 9.00 ± 0.30 MPa (DN) to 19.17 ± 0.36 MPa (GO reinforced carr/PAAm DN (GDN)) and 18.32 ± 0.3 MPa (PDN) at 99% strain, respectively. These results indicate that the introduction of GO and PGO efficiently dissipates the mechanical energy during loading, leading to a 113% and 104% improvement in compressive strength. The mechanical improvement mostly derives from the physical entanglements between soft polymeric chains and rigid two-dimensional GO nanosheets, enhancing energy adsorption during deformation. The compressive strength and toughness of GDN gels were slightly higher than that of PDN gels, which may result from hydrogen bonds between functional groups on the GO nanosheets and amine groups on the PAAm network, improving the cross-linking bonds in the GDN hydrogels.

The fatigue resistance of PDN gels has been systematically investigated by performing cyclic loading-unloading tests at constant strain. Figure 4b,c present the consecutive loading-unloading curves with gradient increases in the maximum strain (30%, 60%, and 90%) of DN and PDN gels, respectively. The compressive strength and hysteresis energy of PDN gels were both largely higher than that of DN gels (Table S2), which also indicates that PGO can efficiently improve the mechanical properties of DN gels. Both DN and PDN hydrogels underwent two sequential loading-unloading cycles (30% and 60% strain), and these loading curves were mostly overlapped, which demonstrates that the carrageenan network is intact under low strain. However, the loading curves of 90% strain deviate below the previous loading curves, which indicates the carrageenan network is damaged. Figure 4d presents five immediate consecutive loading-unloading curves of PDN at 90% compression strain, while DN gels crashed into pieces after the first 90% strain compression. It demonstrates that PDN hydrogels own stronger mechanical properties than DN gels. The PDN gels showed that the hysteresis loops become smaller in subsequent loading. This phenomenon demonstrates that the carrageenan network was damaged under large deformation. The compressive strength at 90% strain of the PDN gel largely decayed from 6.08 MPa for the first loading to 5.48, 5.21, 4.95, and 4.72 for the second, third, fourth, and fifth runs. To further investigate the internal fracture and fluid pressurization behavior of PDN hydrogels, the swelling measurements of compression-tested hydrogels were presented in Figure 4e. These PDN hydrogels showed an increasing swelling ratio over time until equilibrium. In PDN hydrogels, the damaged carrageenan fragments linked with PAAm chains afford a robust osmotic pressure to swell the compression-tested PDN hydrogels, leading to an increased swelling ratio from 179.4 ± 17.9% to 253.9 ± 2.5% after one compression cycle and 284.1 ± 12.3% after two compression cycles at equilibrium state. This result indicates that the brittle carrageenan network is damaged under compression, which is consistent with the mechanical results. Cyclic loading-unloading measurements were also performed at predefined strains (30% and 60%) of DN and PDN hydrogels (Figure S2 and Table S3). With increasing cycles, the compressive strength and hysteresis energy at these predefined maximum strains all depicted a slight decay. Figure 4f presents the representative fifth cycle loop with different maximum strains (30%, 60%, and 90%) of PDN gels. The loading curves with increasing strain overlapped with the previous unloading curves. This observation indicates that PDN hydrogels possess poor fatigue resistance that is similar to other DN hydrogels [43,44]. Therefore, developing an efficient method to fully or partially recover the mechanical property of tough DN hydrogels after compression, is urgent.

### 3.3. Thermal Recovery

**Figure 5:**
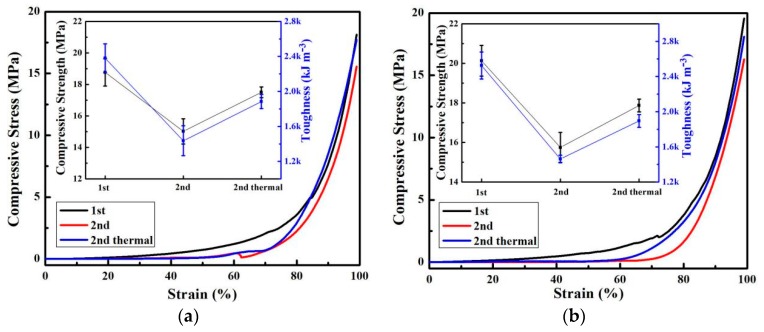
Thermal recovery of the compressive properties of (**a**) PDN hydrogels and (**b**) GDN hydrogels.

To determine the optimized temperature of the gel-sol transition for the DN gels, DSC was used. Figure S3 shows the DSC curve of carrageenan, indicating that the sol-gel transition temperature of carrageenan was around 60 °C. Both compressed GDN and PDN hydrogels were heated around 60 °C to investigate their thermal recovery ability (Figure 5). After the first compression, the compressive strength and toughness of PDN hydrogels largely decreased due to the breakage of the carrageenan network. The PDN hydrogels could only present 79.00% and 60.38% of the compressive strength and toughness on the second compression, respectively. After thermal treatment, most of the compressive strength (94.88%) and toughness (80.85%) were recovered. Figure 5b shows a similar trend of GDN hydrogels after thermal treatment. These results indicate that the thermal treatment can effectively recover the mechanical properties of GDN and PDN hydrogels by rebuilding a brittle carrageenan network. Although both GDN and PDN presented excellent recovered mechanical properties after thermal treatment, the thermal treatment involved direct heating, making it difficult to be implemented within the human body. In this case, developing a photothermal method to recover the mechanical properties of DN hydrogels is necessarily required.

### 3.4. Photothermal Recovery

**Figure 6:**
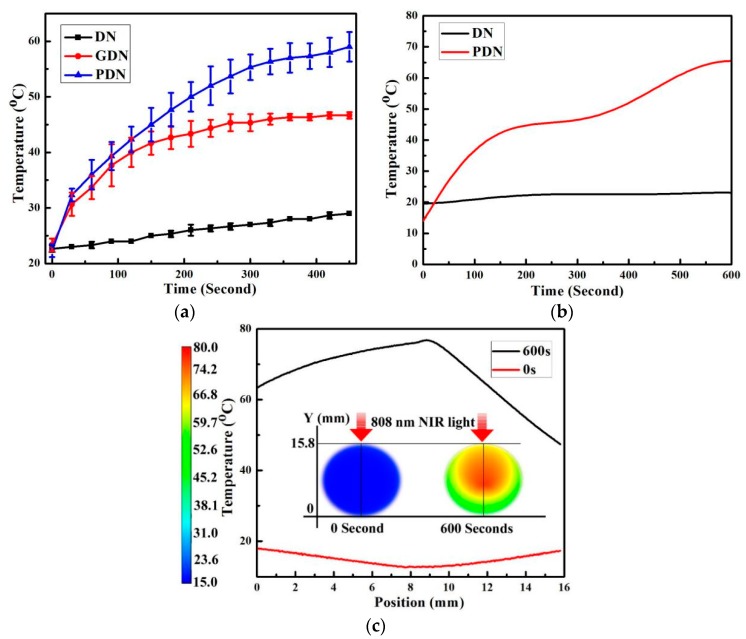
(**a**) Temperature change of DN, GDN, and PDN hydrogels with NIR laser irradiation (808 nm, 2.5 W cm^−2^) detected by a thermometer, (**b**) fitted curve of average temperature of DN and PDN hydrogels with NIR laser irradiation (808 nm, 2.5 W cm^−2^) for 10 min detected by a high-precision digital thermometer (Omega), and (**c**) infrared images of temperature distribution in DN and PDN gels with NIR laser irradiation (808 nm, 2.5 W cm^−2^) for 600 s.

The PGO was added as reinforcing nanofiller and photothermal agent to improve the mechanical properties of hydrogels and gift the function of precise, specific, and human-friendly heating. As the heating only occurred on the hydrogels, limited damage could happen to the surrounding tissue, which is very suitable for clinic application. The NIR photothermal effect of PGO in PDN hydrogels was systematically investigated. Figure 6a shows the temperature change of DN, GDN, and PDN hydrogels with the 808 nm laser irradiation (2.5 W cm^−2^) for 450 s. No big temperature change was found in DN gels because of a lack of photothermal materials. The temperature of GDN increased from room temperature to ~45 °C within 200 s. However, after the temperature reached 45 °C, no temperature increase was found even by extending irradiation time. The low temperature could not afford enough heat energy for the gel-sol transition of carrageenan to rebuild the damaged network. However, the temperature of PDN could increase to around 60 °C after 360 s under laser exposure, which demonstrates that PGO can efficiently absorb and convert NIR light into thermal energy. These results also indicate that the PDN hydrogels may have the ability to recover its mechanical properties by NIR laser irradiation.

The penetration depth was also studied in DN and PDN gels. Figure 6b presents the average temperature changes of the cylindrical DN and PDN gels’ top surface under an 808 nm laser with a power density of 2.5 W cm^−2^ for 10 min. The temperature of DN gels was barely changed under the NIR laser, which implies that no materials in DN gels have the ability to absorb and convert the NIR light to heat. Whereas, the temperature of PDN gels was remarkably enhanced in the first 180 s, which indicates that the PGO possesses the ability to absorb the NIR light and transfer light energy into thermal energy to increase the temperature of PDN gels. In the following 180 s, the temperature of PDN gels was almost steady, which illustrates that the gel-sol transition in PDN gels absorbs a large amount of thermal energy. After that, the temperature of PDN gels was continuously increased in the following 240 s, which showed that the gel-sol transition of PDN gels was completed. Finally, the temperature flattened again. This is because large amounts of heat energy are transferred to the surroundings due to thermal convection, conduction, and irradiation. As shown in Figure 6c, the temperature distribution on the top surface of cylindrical PDN gels was presented, and the temperature on the edge of PDN gels was lower than the temperature in the center of PDN gels. Therefore, the PGO effectively converts the NIR light into thermal energy to rebuild the carrageenan network, which may partially or fully recover the mechanical property of PDN gels.

**Figure 7:**
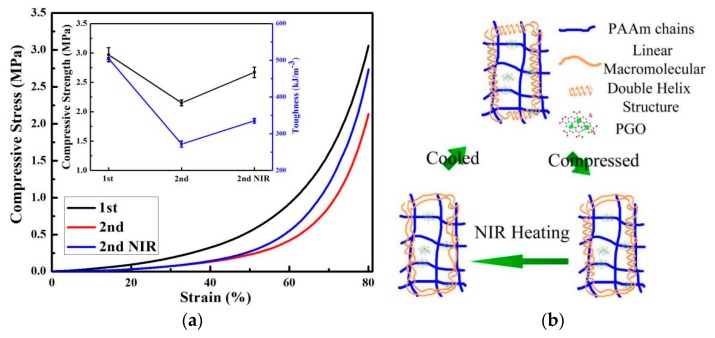
(**a**) The compressive stress-strain curves of PDN gels at 1st compression, 2nd compression, and 2nd compression with NIR laser. The insert is the comparison of their toughness and compressive strength. (**b**) Schematic diagram of NIR photothermal recovery of PDN hydrogels.

Figure 7a presents the compressive stress-strain curves of PDN gels at the first compression, second compression, and second compression with NIR laser irradiation. After NIR laser irradiation, the compressive strength and toughness were clearly increased (the insert in Figure 7a) from 73.40% to 89.90%, and from 53.91% to 66.50%, respectively. The recovery efficiency of NIR laser treatment is a little lower than that of the thermal treatment. The reason may be that the edge of the PDN hydrogels had a temperature below 60 °C due to thermal release, leading to unfinished thermal recovery. These results demonstrate that the PDN gels have the ability to partially recover their mechanical properties by NIR laser irradiation. Figure 7b presents a schematic diagram of the NIR photothermal recovery process. The PGO nanosheets work as heating sources by absorbing and converting light energy into thermal energy. The broken carrageenan network is rebuilt by absorbing thermal energy deriving from PGO. After cooling, the mechanical properties of PDN are recovered.

**Figure 8:**
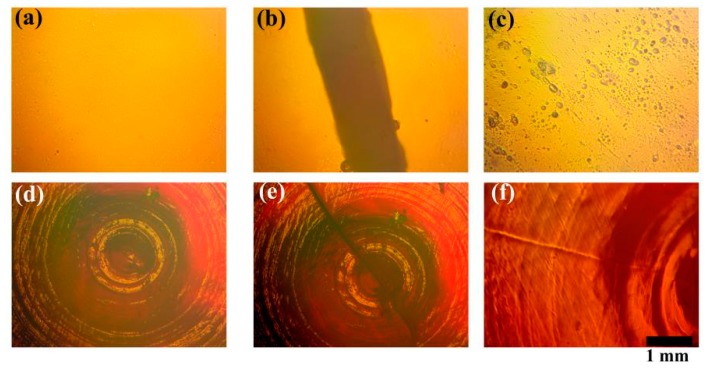
Microscope images of as-prepared (**a**) carrageenan gels and (**d**) PDN gels, cut (**b**) carrageenan gels and (**e**) PDN gels, and healed (**c**) carrageenan and (**f**) PDN gels.

Figure 8a–c present microscopic images of the healed surface of carrageenan gels after thermal treatment, which demonstrate that carrageenan gels can be reformed by thermal-melting. Figure 8e depicts the surface of the PDN hydrogels with a crack by a knife. After 808 nm laser irradiation, the crack was healed with a small scar on the PDN gels’ surface. These results demonstrate that the PDN hydrogels can be healed by NIR laser irradiation due to the presence of PGO. Therefore, these photothermal reversible and tough hydrogels were successfully developed, which may be employed as promising substitutes in load-bearing tissues.

## 4. Conclusions

In summary, biocompatible porphyrin decorated graphene oxide (PGO) reinforced carrageenan/polyacrylamide double network hydrogels (PDN) were first developed. With the introduction of PGO, the compressive strength of PDN was improved at 104% compared to that of carrageenan/polyacrylamide double network hydrogels (DN). In addition, the fatigue resistance of PDN was systematically investigated, which indicated that the PDN possesses poor fatigue resistance similar to other previous double network hydrogels. After near-infrared (NIR) laser irradiation, around 90% compressive strength was recovered, which demonstrates that the PGO efficiently absorb and convert NIR light energy into thermal energy and the carrageenan network is rebuilt during the photothermal and cooling period. Therefore, by introducing PGO into DN hydrogels, the PDN hydrogels possess photothermal recovery and superior mechanical properties, which may find applications as promising substitutes for load-bearing tissues.

## Supplementary Materials

The following are available online at https://www.mdpi.com/2079-4991/9/10/1487/s1, Figure S1: Zeta potential of (a) GO and (b) PGO, Figure S2: Representative cyclic loading−unloading curves of (a) DN and (b) PDN hydrogels for up to five cycles at 30% and 60% strain compression, Figure S3: DSC curves of carrageen, Table S1: Compressive properties of tough hydrogels with a maximum strain at 99%, Table S2: Compressive Properties of tough hydrogels with different strain, Table S3: Cyclic compressive properties of tough hydrogel with different strain.
