# Supplementary material for: Thermal-Recoverable Tough Hydrogels Enhanced by Porphyrin Decorated Graphene Oxide
Source: Nanomaterials (Basel). 2019 Oct 18;9(10):1487. doi: 10.3390/nano9101487 (PMC6835457; doi:10.3390/nano9101487)
Supplement: Supplementary file 1 [file nanomaterials-09-01487-s001.pdf]

Supplementary Materials

Thermal-Recoverable Tough Hydrogels Enhanced by Porphyrin Decorated Graphene Oxide

Jilong Wang [1], Junhua Wei [2,*], Siheng Su [3], Jingjing Qiu [2,*], Zhonglue Hu [4], Molla Hasan [5], Evan Vargas [2], Michelle Pantoya [2] and Shiren Wang [6]

1 Key Laboratory of Textile Science & Technology of Ministry of Education, College of Textiles, Donghua University, Shanghai 201620, China; jilong.wang@dhu.edu.cn
2 Department of Mechanical Engineering, Texas Tech University, 2500 Broadway, P.O. Box 43061, Lubbock, TX 79409, USA; evan.vargas@ttu.edu (E.V.); michelle.pantoya@ttu.edu (M.P.)
3 Department of Mechanical Engineering, California State University at Fullerton, Fullerton, CA 92831, USA; ssu@fullerton.edu
4 College of Engineering, Zhejiang Normal University, Jinhua 321000, China; zhonglue.hu@zjnu.edu.cn
5 Inamori School of Engineering, Alfred University, Alfred, NY 14802, USA; hasanm@alfred.edu
6 Department of Industrial and Systems Engineering, Texas A&M University, College Station, TX 77843, USA; s.wang@tamu.edu
* Correspondence: junhua5wei@gmail.com (J.W.); jenny.qiu@ttu.edu (J.Q.)

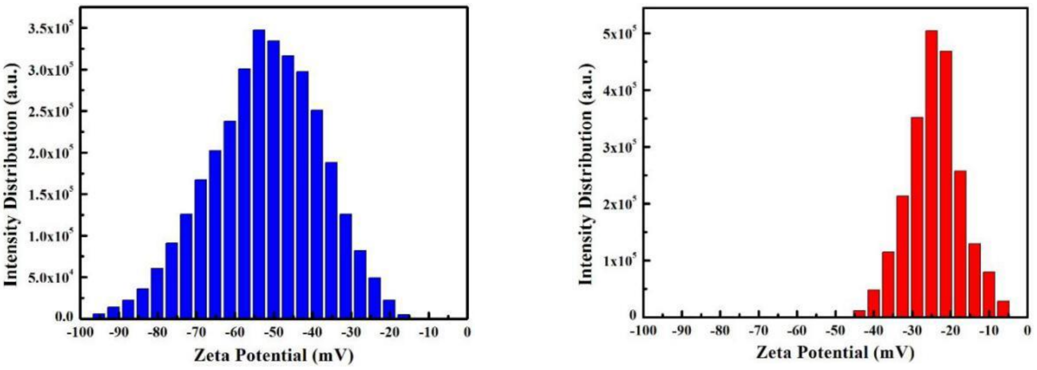

(**a**) (**b**)

Figure S1. Zeta potential of (a) GO and (b) PGO.

Table S1. Compressive properties of tough hydrogels with a maximum strain at 99%.

Sample	Young's Modulus (kPa)	Strength (MPa)	Toughness (kJ m^{-3})
DN	4.85 ± 1.58	9.00 ± 0.30	811.65 ± 59.49
GDN	45.98 ± 11.68	19.17 ± 0.36	2291.01 ± 124.55
PDN	31.69 ± 7.00	18.32 ± 0.30	2140.73 ± 106.94

Table S2. Compressive Properties of tough hydrogels with different strain.

Samples	30% Strength (kPa)	30% Hysteresis Energy (kJ m^{-3})	60% Strength (kPa)	60% Hysteresis Energy (kJ m^{-3})	90% Strength (MPa)	90% Hysteresis Energy (kJ m^{-3})
DN	37.37 ± 2.88	1.73 ± 0.21	222.74 ± 20.27	15.35 ± 2.22	2.50 ± 0.23	207.61 ± 2.88
PDN	114.61 ± 22.27	5.58 ± 1.24	730.86 ± 55.14	80.14 ± 13.56	6.38 ± 0.51	650.28 ± 17.78

Table S3. Cyclic compressive preperties of tough hydrogel with different strain.

Samples	Cycles	30% Hysteresis Energy (kJ m^{-3})	30% Strength (kPa)	60% Hysteresis Energy (kJ m^{-3})	60% Strength (kPa)	90% Hysteresis Energy (kJ m^{-3})	90% Strength (MPa)
DN	1	2.58 ± 0.85	50.70 ± 9.17	20.11 ± 3.16	382.10 ± 27.01	272.24 ± 77.47	3.54 ± 1.18
	2	2.02 ± 0.78	51.45 ± 7.00	15.16 ± 2.87	360.90 ± 22.34	N/A	N/A
	3	1.84 ± 0.71	51.05 ± 6.01	14.83 ± 2.83	346.72 ± 13.46	N/A	N/A
	4	1.78 ± 0.71	50.70 ± 5.09	14.27 ± 2.10	343.35 ± 15.34	N/A	N/A
	5	1.75 ± 0.69	50.85 ± 4.74	14.40 ± 1.77	343.93 ± 13.75	N/A	N/A
PDN	1	5.18 ± 1.31	106.64 ± 20.88	68.73 ± 15.82	705.45 ± 101.04	591.75 ± 95.53	6.08 ± 0.79
	2	4.08 ± 0.84	101.92 ± 20.03	36.77 ± 4.85	668.36 ± 97.45	363.91 ± 50.58	5.48 ± 0.79
	3	3.81 ± 0.76	99.45 ± 19.23	31.88 ± 3.07	634.29 ± 86.55	339.62 ± 48.71	5.21 ± 0.76
	4	3.66 ± 0.72	97.14 ± 19.01	29.69 ± 2.78	607.65 ± 79.59	324.31 ± 40.71	4.95 ± 0.65
	5	3.57 ± 0.56	95.29 ± 18.96	28.86 ± 3.27	589.37 ± 78.08	310.33 ± 38.15	4.72 ± 0.56

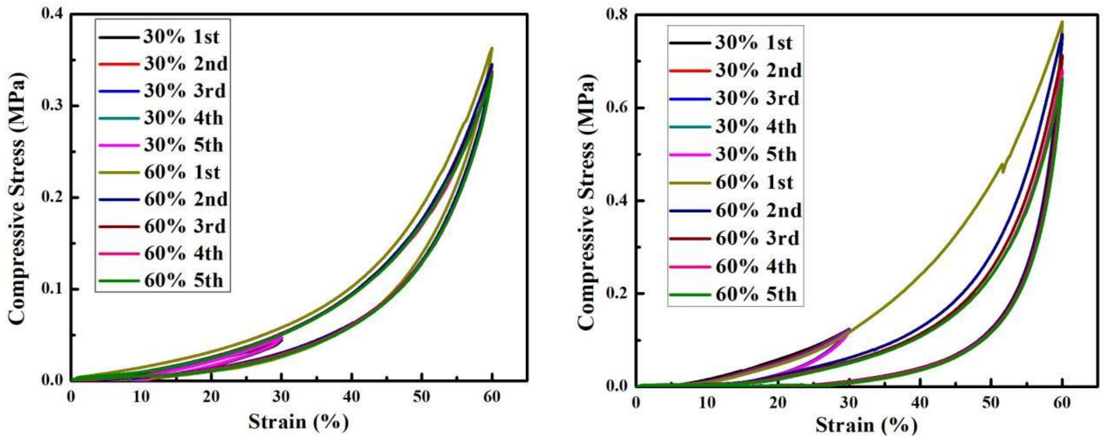

(a) (b)

Figure S2 Representative cyclic loading–unloading curves of (a) DN and (b) PDN hydrogels for up to five cycles at 30% and 60% strain compression.

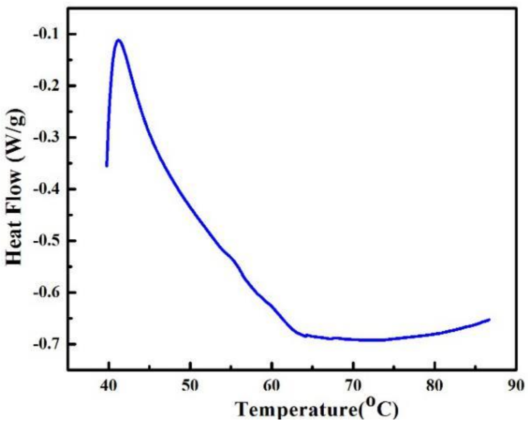

Figure S3 DSC curves of carrageen.
